# The effect of urban–rural resident basic medical insurance on physical health of the rural older adult in China

**DOI:** 10.3389/fpubh.2024.1319697

**Published:** 2024-01-26

**Authors:** Xiaohong Pu, Sichang He, Xi Lin

**Affiliations:** School of Public Administration, Sichuan University, Chengdu, China

**Keywords:** urban–rural resident basic medical insurance, rural older adult, activities of daily living, CLHLS, China

## Abstract

**Introduction:**

Urban–Rural Resident Basic Medical Insurance (URRBMI) is an important system for effectively transferring disease risks to the rural older adult. As China experiences rapid aging, maintaining the physical health of the rural older adult is key to achieving the goal of healthy aging.

**Methods:**

The study explores the impact of URRBMI on physical health of the rural older adult in China using the Chinese Longitudinal Healthy Longevity Survey (CLHLS) data in 2018. Ordinary least square models were used to analyze the relationship between URRBMI and physical health of the rural older adult, and we used instrumental variable method to address the potential endogenous problem.

**Results:**

We find that URRBMI greatly improves physical health of the rural older adult. The heterogeneity analysis indicates that URRBMI contributes more significantly to the rural older adult in eastern areas and the advanced rural older adult. The results also suggested that URRBMI improves physical health of the rural older adult through increasing life satisfaction and enhancing the timeliness of medical services.

**Recommendations:**

This study implies that we need to further improve the participation rate, increase the actual reimbursement ratio and increase financial subsidies for URRBMI in central and western areas, and further integrate the distribution of medical resources to promote physical health of the rural older adult.

## Introduction

Population aging has become increasingly serious in all countries of the world, increasing life expectancy and changes in the disease spectrum have led to a worrying health status for the older adult. The older adult, especially rural older adult, as a vulnerable health group in the population as a whole (1), they generally face more serious disease risks, we should put more emphasis on the physical health of the rural older adult (2). In order to spread the disease risks, many countries around the world have established medical insurance schemes (3, 4). China began to integrate Urban–Rural Resident Basic Medical Insurance (URRBMI) based on the Urban Resident Basic Medical Insurance and the New Cooperative Medical Scheme in 2016. The primary goal of URRBMI is to decrease the medical costs, promote the utilization of medical services and contribute to better health. The physical health of the rural older adult contributes to their life quality and well-being, and is also strongly linked to healthy aging. With the deepening aging, we must pay attention to the rural older adult and discover the healthy function of URRBMI for the rural older adult. Thus, it is an essential topic to discuss whether URRBMI affects physical health of the rural older adult in China, this will help improve URRBMI policy and further enhance its healthy effects.

The Chinese government has always placed the protection of people’s health as a strategic priority for development, and has continuously improved its policies for the promotion of people’s health. Achieving the goal of “Healthy China” means that the achievements of development must benefit all residents fairly (5), especially the vulnerable and high-risk groups, ensuring that “no one is left behind.” Aging is an important demographic feature in China (6, 7). The aging problem is especially serious in rural China. According to the statistical data, China’s rural population aged over 60 has reached 121 million, and the rural population aging at 23.81% (8), the scale of the rural older adult in China is huge. The number of older adult with limitations in Activities of Daily Living (ADL) will increase to 37.3 million in 2050 (1), and their demand for medical insurance is increasing.

Previous studies have examined the relationship between basic medical insurance and health status but have come to different conclusions. Several scholars have stated that basic medical insurance positively affects health status (9–11), because medical insurance can reduce the price of medical services (12), offer more opportunities for medical care and higher-quality health services (13). However, other researchers have shown that basic medical insurance has little effect on the improvement of residents’ health (14–16), the probable explanation lies in the fact that the current policy only focuses on the most basic issues, the finite reimbursement rates, and the limited protection for the health vulnerable population (17).

Many interesting results have been found on the above issues, but there are still some gaps needed to be filled. Several previous studies have mostly focused on the whole population. So far, however, in a rapidly aging society, there is an insufficient wealth of research dedicated to the health of basic medical insurance for the rural older adult, we are particularly concerned about the rural older adult in China, who are the most vulnerable to illness. Furthermore, most of the previous studies used self-assessed health to measure health status, but self-assessed health is subjective, so this study overcomes the shortcomings of self-assessed health by using ADL to represent the objective health status of rural older adult. Improving the physical health of the rural older adult is an essential task to cope with the healthy aging, we must keep an eye on the rural older adult and explore the role of URRBMI for the rural older adult. It will help enrich the theory of basic medical insurance and the study of healthy aging issues.

This study uses the data from CLHLS in 2018 to explore how URRBMI influences physical health of the rural older adult in China. The article made the following contributions: First, the purpose of the study is to provide new empirical proof for current relevant studies by examining the influence of URRBMI on the physical health of the rural older adult under the context of healthy aging. Second, we discussed the heterogeneous influence of URRBMI on physical health of the rural older adult from the viewpoints of different areas and ages, and offers some critical perspectives for improving the URRBMI in the future. Third, we also discussed the influence mechanism between URRBMI and physical health of the rural older adult.

The remainder of this study is organized as follows. Section 2 proposes the research hypothesis. Section 3 presents the data sources and empirical models. Section 4 presents the empirical results. Section 5 offers the discussion and policy recommendations, and finally, Section 6 offers the research conclusions and limitations.

## Research hypothesis

URRBMI is an essential part of the social welfare scheme in the rural areas. When rural older adult are not enrolled in the URRBMI, they have to pay the full medical costs when they fall ill, and therefore they may choose not to receive treatment, which may be detrimental to their health. After participating in the URRBMI, on the one hand, the price of medical services has been decreased owing to the broaden of URRBMI coverage, and the health of the rural older adult can be promoted by decreasing out-of-pocket costs and enhancing their medical services utilization (18). On the other hand, URRBMI offers the rural older adult with protection against disease (19), it has changed the previous traditional concept of not seeking medical services for illnesses, and increased their motivation to pay attention to their physical health, and their awareness of physical health protection has become stronger and stronger. Therefore, participation in the URRBMI is expected to provide greater protection for the physical health of the rural older adult (20), so we propose the following hypothesis:

*H1*: URRBMI can improve the health status of the rural older adult.

The principle of territorial financing and management of URRBMI in China means that there are obvious regional characteristics in the medical insurance resources actually possessed by each region. Due to the disparity in economic levels among the eastern, central and western areas, there are regional disparities in medical services received by the rural older adult in different regions. The eastern region of China is more economically developed (21, 22), and the financial subsidies invested in URRBMI have also increased (23), so the level of medical insurance coverage is generally better in the eastern region. The advanced medical resources and perfect medical conditions are mainly distributed in the eastern region (24, 25), differences in the access to medical services in the eastern, central and western areas may further widen the gap in the medical insurance benefits for the rural older adult. Generally speaking, URRBMI contributed more strongly to the physical health of the rural older adult in eastern areas. We propose the following hypothesis:

*H2a*: The effect of URRBMI on physical health of the rural older adult in eastern areas is more significant than in central and western areas.

The life cycle theory provides an explanation for the fact that the rate of illness dramatically increases as people grow older (26). As we all know, age plays a fundamental role in the physical health of the older adult. Compared to the advanced rural older adult, younger rural older adult make relatively less use of medical services, because they are younger and their physiological functions have not deteriorated significantly. With increasing age, the health degradation rate of the advanced older adult rises. The advanced rural older adult are generally subject to more disease risks, and their specialized medical services need increases, which means that the advanced rural older adult are more in need of the protection of URRBMI. Wu et al. also found that the medical insurance significantly reduces the mortality risk of the advanced older adult (27). Therefore, there is a possible age difference, and URRBMI has a more obvious promotion effect on the physical health of the advanced rural older adult. We propose the following hypothesis:

*H2b*: The effect of URRBMI on physical health of the advanced rural older adult is more significant than the younger rural older adult.

URRBMI promotes the access to medical services for the rural older adult and is an essential guarantee for meeting the medical demands of the rural older adult. By reimbursing the medical costs of the rural older adult, the disease financial burden has been reduced, thus minimizing the influence of catastrophic medical expenditures on the lives of the rural older adult and reducing to a greater extent the disease risks among the rural older adult. Finkelstein et al. also believes that medical insurance may have a positive effect on health due to the increased financial accessibility of medical care (28). At the same time, URRBMI helps rural older adult to reduce precautionary savings, increase current life consumption expenditures, and alleviate the pressure of life caused by medical care (29), the increase in relative incomes effectively improves life quality of the rural older adult and improve their life satisfaction, thus contributing to the improvement of their physical health.

*H3a*: Life satisfaction mediates the effect of URRBMI on physical health of the rural older adult.

According to the Anderson Health Services Utilization Model (30), timeliness of medical services utilization can improve the physical health of the rural older adult through more specialized medical resources (31). URRBMI, as a public policy to promote health, is beneficial to improving the accessibility of medical services for rural older adult, so that they can be more promptly informed of their own health status and can enhance health awareness of the rural older adult (32), thereby avoiding the expansion of disease risks. Hoffman et al. also believes that medical insurance can further improve people’s health through the accessibility of medical services utilization (33). Therefore, URRBMI can promote the health status of the rural older adult by enhancing the timeliness of medical services utilization. We propose the following hypothesis:

*H3b*: Timeliness of medical services mediates the effect of URRBMI on physical health of the rural older adult.

## Data, variables, and empirical model

### Data

**Figure 1:**
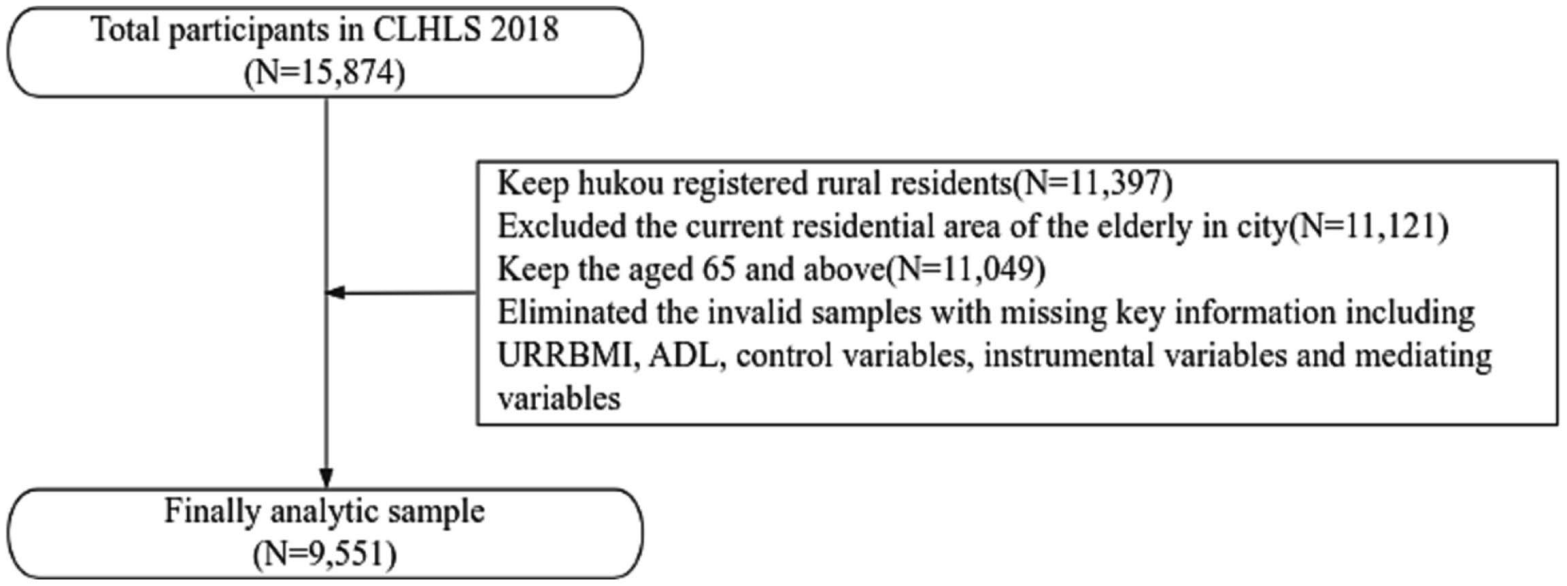
Sample selection procedure of this study.

The study uses the latest data from CLHLS in 2018. CLHLS data is a national, large-scale database for the older adult (34–36), so CLHLS data samples are nationally representative. Besides, the 2018 CLHLS data include detailed variables of URRBMI, physical health of the rural older adult, and timeliness of medical services, and so no, which are the basis for this analysis. According to the purpose of the study, the sample was selected according to the following criteria: to retain the aged 65 and above, have rural household registration and live in rural areas at the time of the survey. Additionally, the invalid samples with missing key information including URRBMI, ADL, gender, age, marriage, education years, smoke, drink, exercise, physical examination, co-residence, life satisfaction, timeliness of medical services and who pays for medical services mainly were eliminated, and the final valid samples is 9,551 (Figure 1).

## Variables

### Dependent variable

The dependent variable is physical health of the rural older adult. We use the ADL to reflect physical health. ADL is objective indicator of physical condition and can indicate the health condition of the rural older adult (37), and referring to the previous literature (38–41), ADL has been widely used to measure physical health, so we use ADL reflect the health status of the rural older adult. ADL was measured by the following items: (1) Bathing; (2) Dressing; (3) Indoor moving; (4) Toileting; (5) Continence of defecation; (6) Eating. Each item was scored from 1 to 3, and the total score of ADL could reveal the health status. The more scores the respondents received, the higher ADL reliance would be, which means they are in poor health (38). At the same time, we use a binary variable defined as ADL-1 that equals 0 if the respondent reported no limitation in six items above, and otherwise equals 1. We take ADL as a key proxy variable physical health of the rural older adult.

### Independent variable

The independent variable is whether the rural older adult participated in URRBMI (*URRBMI*). The rural older adult enrolled in the URRBMI were assigned a value of 1, otherwise 0.

### Control variables

According to the other studies (42–44), we selected the control variables as follows: gender (*Gender*), age (*Age*), marriage (*Marriage*), education years (*Education*), smoke (*Smoke*), drink (*Drink*), exercise (*Exercise*), physical examination (*Examination*), co-residence (*Residence*).

### Instrumental variable

This study uses who pays for medical services mainly (*Expense*) as an instrumental variable, we set *Expense* as 1–3, representing medical insurance payment, pay personally, and others, respectively. We use *Expense* as the instrumental variable, the reasons are as follows: First, whether or not the rural older adult are enrolled in URRBMI is affected by the variable of who pays for medical services mainly, therefore, who pays for medical services mainly affects the willingness of the rural older adult to participate in URRBMI. Second, the variable of who pays for medical services mainly has no direct influence on the physical health of the rural older adult.

### Mediating variable

Life satisfaction (*satisfaction*) and timeliness of medical services (*Service*) may relate to the URRBMI and physical health of the rural older adult, which may affect the relationship between them. Relevant studies demonstrated that timely access to medical services could improve the chances of healthy survival for the older adult (45), and life satisfaction is known to be positively correlated with physical health (46). Referring to the previous literature (39, 47–50), the study selected *satisfaction* and *Service* to examine the mediating effect.

The above variables and their definitions are shown Table 1, and their descriptive statistics are given in Table 2.

**Table 1:** The definitions of all variables.

Variables	Symbols	Definitions
Dependent variable		
Activities of daily living	ADL	*ADL* is equal to 6–18 scale, representing the rural older adult physical health, respectively.
	ADL-1	*ADL-1* is equal to 1 if the rural older adult is restriction in six daily activities, and otherwise 0.
Independent variable		
Urban–Rural resident basic medical insurance	URRBMI	*URRBMI* is equal to 1 if the rural older adult participated in the URRBMI, and otherwise 0.
Mediating variable		
Life satisfaction	Satisfaction	*Satisfaction* is equal to 1–5, representing very good, good, so so, bad, very bad.
Timeliness of medical services	Service	*Service* is equal to 1 if the rural older adult can get timeliness of medical services, and otherwise 2.
Instrumental variable		
who pays for medical services mainly	Expense	*Expense* is equal 1–3, representing medical insurance payment, pay personally, and others.
Control variables		
Gender	Gender	*Gender* is equal 1 if the rural older adult is male, and otherwise 2.
Marriage	Marriage	*Marriage* is equal 1 if the rural older adult is in marriage period, and otherwise 0.
Age	Age	*Age* is 65–117.
Education years	Education	*Education* is 0–16, indicating that education years
Smoke	Smoke	*Smoke* is equal 1 if the rural older adult is smoke, and otherwise 0.
Drink	Drink	*Drink* is equal 1 if the rural older adult is drink, and otherwise 0.
Exercise	Exercise	*Exercise* is equal 1 if the rural older adult is exercise, and otherwise 0.
Physical examination	Examination	*Examination* is equal 1 if the rural older adult have regular physical examination, and otherwise 0.
Co-residence	Residence	*Residence* is equal 0–2, representing alone, with household member, institution.

**Table 2:** Descriptive statistics.

Variables	Obs	Mean	S.D.	Min	Max
ADL	9,551	7.149	2.613	6.000	18.000
ADL-1	9,551	0.206	0.404	0.000	1.000
URRBMI	9,551	0.836	0.370	0.000	1.000
Gender	9,551	1.575	0.494	1.000	2.000
Marriage	9,551	0.375	0.484	0.000	1.000
Age	9,551	85.677	11.695	65.000	117.000
Education	9,551	4.260	5.791	0.000	16.000
Smoke	9,551	0.163	0.369	0.000	1.000
Drink	9,551	0.147	0.354	0.000	1.000
Exercise	9,551	0.239	0.427	0.000	1.000
Examination	9,551	0.692	0.462	0.000	1.000
Residence	9,551	0.841	0.406	0.000	2.000
Expense	9,551	1.572	0.587	1.000	3.000
Satisfaction	9,551	2.248	0.781	1.000	5.000
Service	9,551	1.037	0.188	1.000	2.000

### Empirical model

Referring to the previous literature (43, 51, 52), the regression model is set as follows:


(1) ADLi=β1+β2URRBMIi+β3Controls+εi

where *ADL* refers to the rural older adult physical health; *URRBMI* represents the variable of URRBMI; *Controls* stands for the above control variables, β1 indicates the intercepted item; β2 denotes the coefficient of URRBMI; β3 is the coefficients of control variables; εi is a normally distributed random error vector.

β2 is the coefficient of interest. If β2<0, it means that URRBMI promotes the physical health of the rural older adult. If so, H1 is confirmed. In contrast, if β2>0, it indicates that URRBMI weakens the health status of the rural older adult. If so, according to the research, H1 does not stand.

## Empirical results

### Benchmark regression results

In this section, ordinary least square models were used to analyze the regression of physical health of the rural older adult. The estimated results are shown in Table 3. We see that all the coefficients of *URRBMI* are significantly negative at the 1% level. It means that URRBMI can prompt physical health of the rural older adult and confirm the H1 is right.

**Table 3:** The Benchmark regression result.

Variables	(1)	(2)
URRBMI	−0.302^***^	−0.241^***^
	(0.072)	(0.065)
Gender		0.127^**^
		(0.056)
Marriage		−0.235^***^
		(0.064)
Age		0.0663^***^
		(0.002)
Education		−0.0128^***^
		(0.004)
Smoke		−0.232^***^
		(0.072)
Drink		−0.295^***^
		(0.072)
Exercise		−0.630^***^
		(0.057)
Examination		−0.727^***^
		(0.053)
Residence		0.879^***^
		(0.062)
Constant	7.402^***^	1.607^***^
	(0.066)	(0.273)
Observations	9,551	9,551
*R*-squared	0.001	0.211

***p < 0.01, **p < 0.05; Standard errors are in parentheses (the same below).

For all the control variables, most of the estimates are in agreement with theoretical expectations. Specifically speaking, the coefficients of *Gender, Age, and Residence* are positive at the 5% level, which suggests that the better health of the rural older adult is more apparent among males, younger, and residence alone. Furthermore, at the 1% level, the coefficients of *Marriage, Education*, *Smoke*, *Drink*, *Exercise*, *Examination* is all negative. The findings show that married rural older adult have better physical health status, and education years, regular exercise, regular physical examination can prompt the physical health of the rural older adult.

### Robustness test

In this study, we use the method of replacing the dependent variable for the robustness test, and since ADL-1 is a dummy variable, we adopt a binary logistic regression model to estimate the results. The results are given in Columns (1, 2) of Table 4. The coefficients of *URRBMI* are significantly negative at the 5% level, it means that URRBMI improve the physical health of the rural older adult, this suggests that URRBMI has a protective effect on the physical health of the rural older adult. The outcome is in accordance with the previous results, suggesting that the results keep highly robust and further support the conclusions of this study. The results for the control variables are also in agreement with the above results obtained from the ordinary least square models.

**Table 4:** Regression results of robustness test and endogenous test.

Variables	Robustness test		Endogenous test	
	(1)	(2)	(3)	(4)
URRBMI	−0.158^**^	−0.187^***^		−3.973^**^
	(0.066)	(0.071)		(1.597)
Expense			−0.030^***^	
			(0.006)	
Control variables	No	Yes	Yes	Yes
Constant	−1.218^***^	−0.414^***^	0.872^***^	10.806^***^
	(0.060)	(0.132)	(0.022)	(1.323)
Observations	9,551	9,551	9,551	9,551
Phase|F-value			21.186	
DWH test p*-*value				0.007

***P < 0.01, **p < 0.05.

### Endogenous test

As we all know, there may be a bi-directional causality between URRBMI and the health status of the rural older adult. Generally speaking, rural older adult in poorer health is more inclined to participate in URRBMI, this leads to endogenous problems as the health status of the rural older adult inversely affects the behavior of whether or not to participate in URRBMI (53). Therefore, we solve the endogenous problem using the instrumental variable method (54).

Columns (3, 4) of Table 4 present the estimation outcomes of the endogeneity test. The coefficient of *URRBMI* is still negative, the result is agreement with the previous findings and further demonstrates our conclusion. Compared to not controlling the endogeneity, we also find that the value of the regression coefficients of *URRBMI* decreases after controlling the endogeneity, suggesting that the impact of URRBMI in promoting the physical health of the rural older adult is underestimated if endogeneity is not addressed.

### Heterogeneity analysis

We also investigate the heterogeneous effect from different regions and age. The estimation results are shown in Table 5. Columns (1), (2), and (3) are the results for western, central and eastern areas. Columns (4, 5) are the outcomes of younger rural older adult and advanced rural older adult.

**Table 5:** Estimation results of heterogeneous analysis.

Variables	Region difference			Age difference	
	Western	Central	Eastern	Younger rural older adult	Advanced rural older adult
	(1)	(2)	(3)	(4)	(5)
URRBMI	−0.172	−0.102	−0.314***	−0.022	−0.329***
	(0.123)	(0.143)	(0.099)	(0.050)	(0.103)
Control variables	Yes	Yes	Yes	Yes	Yes
Constant	2.447***	1.475***	1.358***	6.445***	7.876***
	(0.450)	(0.478)	(0.418)	(0.121)	(0.192)
*R*-squared	0.156	0.203	0.254	0.031	0.135
Observations	2,379	2,919	4,253	3,504	6,047

***p < 0.01.

At the 1% level, the *URRBMI* coefficients were significantly negative in the eastern region, but there is no influence on the central and western regions. The influence of URRBMI on the physical health of the rural older adult varies in different regions. Hence, this finding confirmed H2a, the effect of URRBMI on physical health of the rural older adult in eastern areas is more significant than in central and western areas.

Age was classified into two groups, 65–80 years old is considered as the younger rural older adult, and aged over 80 is considered as the advanced rural older adult. Columns (4, 5) in Table 5 present the results, the coefficient of *URRBMI* is significantly negative at the 1% level for the advanced rural older adult, while it is not significant for the younger rural older adult. The result suggests that the URRBMI promotes the health status of the advanced rural older adult, but it has no influence on the younger rural older adult. Hence, this finding confirmed H2b, the effect of URRBMI on physical health of the advanced rural older adult is more significant than the younger rural older adult.

### Mediating effect

Mediated effects analysis can help researchers verify the processes and mechanisms of factor interactions. This study uses Hayes’ identification methodology and test steps to test the mediating effect (55, 56). The model is set as follows:


(2) Mediatori=γ1+γ2URRBMIi+γ3Controls+εi

(3) ADLi=η1+η2URRBMIi+η3Mediatori+η4Controls+εi

where *Mediator* indicates the variable of *satisfaction* or *service*, and the other variables are the same with the Model (1). If *η_2_* and *η_3_* are both significant, it suggests that life satisfaction and timeliness of medical services are partially mediating variables; but if *η_2_* is not significant but *η_3_* is significant, it suggests that life satisfaction and timeliness of medical services are fully mediating variables.

From the results of the Table 6. After adding two mediating variables, life satisfaction and timeliness of medical services, respectively. We can find that the coefficients of life satisfaction and timeliness of medical services were significant at the 1% level, it suggested that URRBMI improves the physical health of the rural older adult through increasing life satisfaction and enhancing the timeliness of medical services, respectively, with both variables playing partial mediating roles, respectively. Hence, this finding confirmed H3a and H3b.

**Table 6:** Results of mediating effect.

Variables	(1)	(2)	(3)	(4)
	Satisfaction	ADL	Service	ADL
URRBMI	0.062^***^	−0.270^***^	−0.016^***^	−0.227^***^
	(0.0213)	(0.0638)	(0.00521)	(0.0644)
Satisfaction		0.468^***^		
		(0.0306)		
Service				0.852^***^
				(0.127)
Control variables	Yes	Yes	Yes	Yes
Constant	2.198^***^	0.579^**^	1.055^***^	0.708^**^
	(0.090)	(0.278)	(0.022)	(0.303)
*R*-squared	0.034	0.230	0.012	0.215
Observations	9,551	9,551	9,551	9,551

***P < 0.01, **P < 0.05.

## Discussion and recommendations

As an important medical security system design in China, URRBMI undertakes a number of missions such as ensuring health rights and safeguarding health justice (57). The study indicated that URRBMI is consistent with the fundamental goal of improving people’s health.

(1) In this study, we found that URRBMI can prompt physical health of the rural older adult. The results are consistent with the previous studies: medical insurance can significantly improve the health status of the older adult (58–61). As we all know, URRBMI can effectively reduce the medical expenditures, and increase the probability that rural older adult have access to higher-quality medical services which prompts the physical health of the rural older adult (62). Besides, with increasing age, the physical functions of the rural older adult deteriorate and their physical health gets worse. The study also suggested that marriage is a protective factor for physical health of the rural older adult, this is consistent with the findings of Fuhrer’s study (63). Regular exercise helps strengthen the immune system and thus reduces the likelihood of disease, so rural older adult who exercise regularly are in better physical health.

Due to the positive physical health implication of URRBMI on the rural older adult, it is quite necessary to further improve its participation rate (64). We should improve the design of URRBMI policy. Continuously expanding the coverage of URRBMI is a precondition for promoting the improvement of physical health of the rural older adult. Expansion of medical insurance coverage significantly increases medical services utilization (65). Particularly, it is difficult to ensure the sustainability of broad coverage due to the current policy of voluntary participation. To further broaden the wide coverage of URRBMI, consideration could be given to compulsory participation in the URRBMI for rural older adult.

(2) The study’s results suggested that URRBMI contributes more significantly to the rural older adult in eastern area and the advanced rural older adult. Because the sophisticated and high-quality health resources are largely distributed in the eastern region (24), rural older adult in eastern area enjoys a higher level of URRBMI, release higher medical demand and can gain medical services more easily (66). However, the economic development is slower in the central and western areas. Even though the URRBMI has increased the demand for medical services by the rural older adult, the demand for medical services still cannot be met within the constraints of the existing medical conditions, so the promotion of URRBMI is not significant. Furthermore, as age increases, the physical health of the advanced rural older adult deteriorates and they need to consume more medical services to maintain their health (67), and the frequency and intensity of the advanced rural older adult use URRBMI is higher than that the younger rural older adult.

Due to the disparity in economic levels between the eastern, central and western areas, there are regional differences in the impact of URRBMI on the physical health of the rural older adult. We should narrow the basic medical insurance compensation gap among eastern, central and western areas. Specifically, we should continue to increase financial subsidies for URRBMI in the central and western areas, and gradually raise the overall level of URRBMI and minimize regional disparities. Reducing regional disparities in URRBMI reimbursement contributes to achieve the regional equalization of basic medical insurance services. Meanwhile, generous insurance reimbursement can decrease the price of medical services (68), it is particularly important to promote the physical health of the advanced older adult. We should maintain the balance of income and expenditure of the medical insurance fund, reduce the threshold line, raise the ceiling line, and increase the actual insurance reimbursement rate to alleviate the financial burden of illness for the rural older adult, especially provide more precise safeguards for the advanced rural older adult.

(3) The results also showed that URRBMI improves physical health of the rural older adult through increasing life satisfaction and enhancing the timeliness of medical services. On the one hand, URRBMI has reduced the burden of medical expenses on the rural older adult and relatively increased their regular income, thus the rural older adult can spend more of their income on such areas as daily leisure consumption and preventive health care, and their quality of life has improved accordingly, which in turn has increased their life satisfaction and improved their physical health. On the other hand, timeliness of medical services shortens the time to acquire medical services, enhances the availability of medical services for rural older adult, thus enhancing their physical health.

To increase the timely access to medical services for rural older adult, we should further integrate the distribution of medical resources. By optimizing the integration of medical resources, improving the efficiency of medical resources allocation, thus forming a reasonable and orderly pattern of access to medical services (69), which significantly improves spatial accessibility of medical services. It will help rural older adult obtain various types of medical services close to their homes, which could gain medical services immediately when they need it.

## Conclusion and limitation

Using the CLHLS data in 2018, this study analyzed the influence of URRBMI on the health status of the rural older adult. We have come to the following conclusions:

First, the URRBMI greatly improves physical health of the rural older adult, and the results are robust. Second, there are regional and age differences in the impact of URRBMI on the physical health of the rural older adult. URRBMI plays a more vital role in prompting physical health of the rural older adult in the eastern area. Furthermore, compared with the younger rural older adult, the effect of URRBMI on improving the physical health of the advanced rural older adult is more obvious. Third, we provide extra evidence that life satisfaction and timeliness of medical services plays a mediating effect in the association between URRBMI and physical health of the rural older adult in China. The compensation mechanism of URRBMI has relatively lowered the price of medical services and enhanced the leisure consumption for the rural older adult, thereby increasing life satisfaction and promoting physical health, and URRBMI guarantees timely access to medical services for the rural older adult, which prevents minor illness from become serious ones (70).

However, there are following limitations in our study and further research is needed. First, the impact of chronic diseases on ADL may be significant, this could affect the analysis results. However, due to the incompleteness of the chronic disease data, chronic disease was not included as a control variable in this study. Second, as the research object of this study is the rural older adult and the relationship between URRBMI and their physical health, this study did not include the urban older adult, the comparison of the two groups could be studied as a new topic in the future.

## Data availability statement

The original contributions presented in the study are included in the article/supplementary material, further inquiries can be directed to the corresponding author.

## Author contributions

XP: Conceptualization, Writing – original draft. SH: Data curation, Software, Writing – original draft. XL: Supervision, Writing – review & editing.
